# A Practical Approach to Management of Acute Pancreatitis: Similarities and Dissimilarities of Disease in Children and Adults

**DOI:** 10.3390/jcm10122545

**Published:** 2021-06-08

**Authors:** Zachary M. Sellers, Monique T. Barakat, Maisam Abu-El-Haija

**Affiliations:** 1 Department of Pediatrics, Division of Pediatric Gastroenterology, Hepatology, and Nutrition, Stanford University, Palo Alto, CA 94304, USA; zsellers@stanford.edu (Z.M.S.); mbarakat@stanford.edu (M.T.B.); 2 Department of Medicine, Division of Gastroenterology and Hepatology, Stanford University, Palo Alto, CA 94304, USA; 3 Division of Pediatric Gastroenterology, Hepatology and Nutrition, Cincinnati Children’s Hospital Medical Center, Cincinnati, OH 45229, USA; 4 Department of Pediatrics, College of Medicine, University of Cincinnati, Cincinnati, OH 45221, USA

**Keywords:** acute pancreatitis, management, outcomes, gastrointestinal disease

## Abstract

Acute pancreatitis (AP) is associated with significant morbidity and mortality, and it substantially contributes to the healthcare burden of gastrointestinal disease and quality of life in children and adults. AP across the lifespan is characterized by similarities and differences in epidemiology, diagnostic modality, etiologies, management, adverse events, long-term outcomes, and areas in greatest need of research. In this review, we touch on each of these shared and distinctive features of AP in children and adults, with an emphasis on recent advances in the conceptualization and management of AP.

## 1. Introduction

Acute pancreatitis (AP) is characteristically a painful, inflammatory disease that targets the exocrine pancreas, but it can have also have a profound impact on the endocrine pancreas and extra-pancreatic tissues. Irrespective of pancreatitis etiology, once pancreatitis develops, the pathophysiology of AP and its mechanism for causing systemic manifestations is similar. Intra-acinar activation of pancreatic enzymes such as trypsin, phospholipase A2, and elastase occurs, resulting in autodigestion and injury to the pancreas. In addition to local damage to the pancreatic parenchyma, these activated enzymes can damage regional tissue and activate the complement system and the inflammatory cascade, producing cytokines and causing profound systemic inflammation and compromising organ function [1,2]. AP impacts both children and adults, yet there is often an unfamiliarity with what is similar and what is different, given they are often managed by different types of providers. To bridge this divide, we have assembled a comparison of the primary similarities and differences between pediatric and adult AP using peer-reviewed manuscripts published in English and indexed in PubMed, with a particular emphasis on recent works. This content is meant to be a practical comparison that is easily accessible to clinicians and researchers, rather than an exhaustive academic analysis.

## 2. Epidemiology

**Figure 1:**
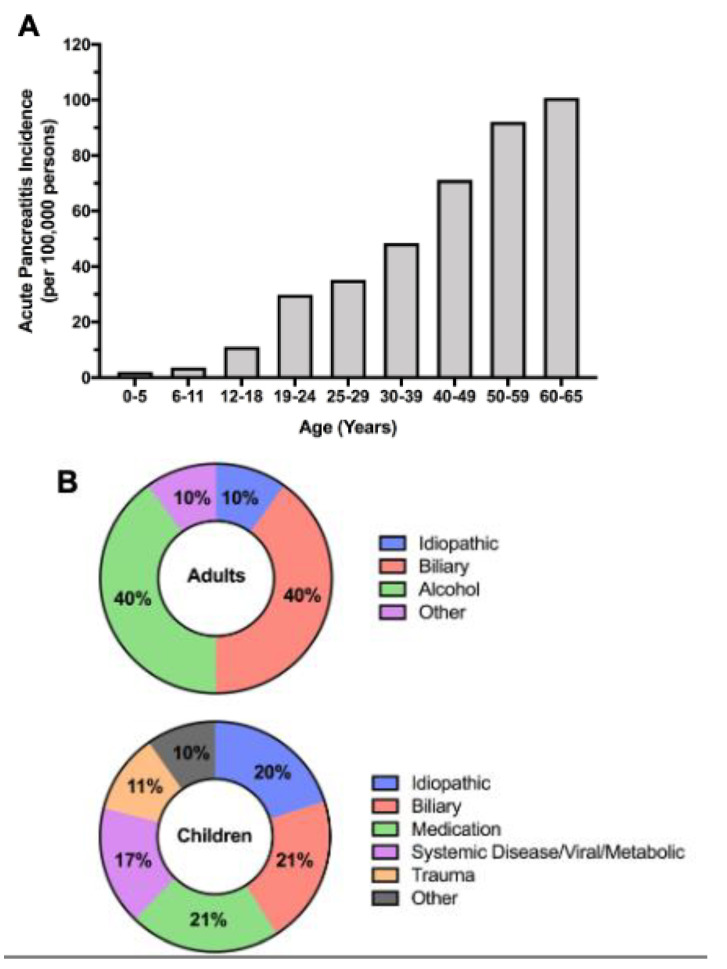
Comparison of the epidemiology and etiologies of pediatric and adult acute pancreatitis (AP). (**A**) Incidence of AP across age groups in 2014 in the United States from the Truven Health MarketScan Commercial Claims and Encounters database. Data represent a composite of both inpatient and outpatient encounters. Adapted from Sellers et al. [7] (**B**) Relative comparison of the differing contributing factors to pediatric and adult AP. Adapted from Abu-El-Haija et al. [10,11] and Husain et al. [12].

Globally, AP is the most common pancreatic disease, reported to occur in 34/100,000 people [3]. There is, however, variation based on geographic location and ages. It should also be noted that epidemiological reports will also differ based on the source of data. With AP as the third most common gastrointestinal cause for hospitalization in the United States [4], only using inpatient data may skew results. Approximately 35% of patients with AP (children or adults) presenting to Emergency Departments will be discharged without admission [5,6]; thus, outpatient encounters are also important in estimating the true incidence of AP. A number of pediatric studies have reported that pediatric AP incidence is “approaching” that of AP in adults; adult AP likely occurs approximately 4–10× more frequently than pediatric AP [7]. The incidence of adult AP is ~40–110/100,000 persons, whereas the incidence of pediatric AP is ~6–12/100,000 persons. Epidemiological trends may also differ between pediatric and adult AP. Looking across several decades, both pediatric and adult AP have increased; however, in isolating analysis to the last decade, AP in adults in the United States has not only stabilized, but may actually be decreasing. The incidence of AP in children does not appear to be decreasing, but it does appear to have stabilized [7,8,9]. Differences in pediatric and adult AP epidemiology may be determined by a number of factors, but certainly, they are highly influenced by differences in etiologies that cause AP (discussed below). Figure 1 summarizes epidemiology patterns of AP.

## 3. Diagnosis

In both children and adults, AP is defined by meeting two out of the three criteria: (i) abdominal pain and symptoms suggestive of pancreatitis, (ii) lipase and/or amylase at least three times the upper limit of the normal range for the laboratory values used, and (iii) imaging findings of AP [13,14]. It is worth noting that consensus definitions of childhood AP were not established until 2012 and endorsed by the North American Society for Pediatric Gastroenterology, Hepatology, and Nutrition (NASPGHAN) in 2017 [14,15]. While biochemical and imaging evidence of AP is similar between children and adults, AP symptoms may differ between children and adults, especially in young children. In examining presenting symptoms of AP in children, back pain occurred in 14% of adolescents, but only 1% of children 1–10 years old. Rather, one of the most common presenting symptoms in 1–10 year-olds (beyond abdominal pain) was fever (~40%) [16,17].

Diagnosis may be similar between children and adults; however, severity classification differs. In adult AP, the revised Atlanta classification is used for defining severe AP. The majority of cases of adult AP are categorized as mild (60–75%), 20–30% as moderate, and 5–10% as severe. The overall mortality is up to 6% [18], but mortality can be as high as 50% in severe AP [18,19,20]. NASPGHAN published a pediatric-focused consensus definition of mild, moderately severe, and severe AP in pediatrics for the first time in 2017 [15]. In this definition, mild AP has been defined as a self-limited phenomenon, whereas moderately severe AP involves local pancreatic complications and/or transient (<48 h) organ dysfunction, and severe AP involves persistent organ dysfunction lasting longer than 48 h. Pediatric-based definitions of multi-organ failure and complications were utilized to define a pediatric classification system. Based on this definition, the majority of children experience mild AP, with 15–34% developing moderately severe and severe AP, with attendant morbidity and mortality [21,22,23].

Reaching the stage of unified definitions for severity classification will allow comparative effectiveness studies to be conducted within the field of AP. Different severity scoring systems have been proposed in adult and pediatric studies in AP to risk stratify patients into predicted severe or non-severe courses [24]. What is needed is a bedside, easy-to-apply scoring tool for risk stratification of patients on admission, and that continues to be a work in progress for both adult and childhood AP.

## 4. Etiologies

AP risk factors differ across one’s lifespan. In adults, the vast majority of AP episodes are associated with excessive alcohol intake or gallstone disease [25]. Of note, recent evidence suggests that alcohol is more likely a risk factor, rather than a cause, of AP [26]. In children, risk factors for and etiologies of AP are more diverse, with up to 20% of pancreatitis episodes attributable to more than one contributing etiology. Up to one-third of children may have no known identifiable cause for pancreatitis. Of those with a known etiology, these are predominated by obstructive biliary causes and medication-related adverse effects [10]. Similar to adults, other causes include hypertriglyceridemia, trauma, viral, metabolic, and endoscopic retrograde cholangiopancreatography (ERCP) [10,12,27,28]. Genetic causes of AP are reported to be low; however, it is not standard practice to perform genetic testing in children with a single episode of AP. Over 35% of acute pancreatitis episodes have been attributed to idiopathic AP, a diagnosis of exclusion of the typical etiologies of AP. With increasingly thorough and systematic evaluations to identify the etiology of AP, rates of idiopathic AP have decreased over time, from 41% in 1998 to 30% in 2007 [29]. Some of these episodes of idiopathic AP may, in fact, represent underlying genetic sensitization for the development of AP in the setting of subclinical triggers for AP. In some series, children have been found to have higher rates of idiopathic AP relative to adults. Research in this area may shed light on the role of genetics in both pediatric and adult AP. Despite some differences in etiology, the pancreatitis disease burden and its impact on quality of life are remarkably similar in children and adults [28]. Figure 1 summarizes etiologies of AP.

## 5. Management of Acute Pancreatitis

Despite many differences in etiology of AP between children and adults, management of AP is very similar. In 2018, NASPGHAN and the American Gastroenterology Association (AGA) issued separate reports on management recommendations for AP [30,31]. Both sets of recommendations set intravenous fluids, early enteral nutrition, and pain control as the foundational pillars of AP management, as well as addressing the use of endoscopy and prophylactic antibiotics. We encourage all pediatric and adult providers to familiarize themselves with these reports to provide equitable care to all individuals with AP. This is important throughout the course of AP, but it is especially important on initial presentation. Given overall less familiarity with pediatric pancreatitis and an increasing hesitation to use opiates, or high rates of intravenous fluids, NASPGHAN recently produced a Pediatric Pancreatitis Passport to facilitate timely and evidence/expert-based AP treatment for those with an established history of pancreatitis [32]. Additional resources, such as admission order sets and online algorithms, also help provide an appropriate standard of care [33].

With the exception of biliary and hypertriglyceridemia-induced pancreatitis, there is effectively no distinction in the management of AP based on etiology, currently. This is, in part, due to limited knowledge about differential pathophysiological processes between etiologies. Given the current viewpoint of shared disease models of inflammation and recovery, treatment recommendations are equivalent. However, emerging data on how drugs [34,35] and ERCP [36,37] induce pancreatic inflammation and affect tissue regeneration are highlighting new ways in which we may be able to design etiology-specific AP risk models as well as treatments rather than a one size fits all approach [38].

## 6. Course and Complications of Acute Pancreatitis

ERCP is well-established as an endoscopic therapeutic modality for the management of biliary and pancreatic disorders in the adult population [39,40,41], with approximately 600,000 ERCPs performed in the United States, annually [42,43]. With gallstone disease being the leading cause of AP in adults, risk stratification protocols have evolved to define the role of ERCP in these patients. The most recent AGA guidelines [31] recommend avoidance of ERCP in patients with gallstone AP, unless there is evidence of biliary obstruction or cholangitis, due to concern that ERCP and potential post-ERCP pancreatitis could accentuate the severity of AP. Given the potential for spontaneous biliary stone passage in patients with AP, imaging is typically undertaken to confirm the presence of a stone prior to ERCP.

ERCP in children is performed less frequently, but it is being established as a modality for management of pancreatic and biliary disease [44], with utilization and outcomes following ERCP increasingly studied and described [45]. There are, to date, no independent studies focused on the role of ERCP in pediatric patients with AP; however, based on the extrapolation of guidelines in the adult population, ERCP is often performed in pediatric patients with AP and evidence of biliary obstruction or cholangitis. In children, therapeutic ERCP for gallstone AP may be delayed relative to adults due to limitations in local pediatric therapeutic endoscopy expertise and need to transfer the patient to another pediatric hospital or engage adult endoscopists to perform the procedure [44].

For both children and adults, endoscopic ultrasound (EUS) or magnetic resonance cholangiopancreatography (MRCP) is typically performed prior to ERCP when laboratory studies and clinical evolution are suggestive of a possible stone resulting in obstruction and/or cholangitis. In children and adults with severe AP, pancreatic fluid collections (pseudocysts) and/or walled-off pancreatic necrosis may develop. In prior decades, this was managed by percutaneous drainage or surgical debridement. The current standard of care for management of these pancreatic collections in adults is EUS-guided drainage of the fluid collection with plastic or lumen apposing metallic stents (LAMS) when the collection is mature [46,47,48]. Acute pancreatitis without necrosis typically does not result in severe complications in children, and severe necrotizing pancreatitis is less common in children relative to adults [28,49]. For children in whom necrotizing pancreatitis develops and results in a persistent collection, placement of LAMS followed by necrosectomy to clear necrotic debris from the cavity is typically performed. Expertise and experience in therapeutic EUS are more limited in pediatric endoscopy, yet EUS-guided drainage is increasingly performed and reported in children with pancreatic fluid/necrotic collections, with favorable outcomes similar to those reported for adults [48,50,51,52]. Studies comparing plastic stents versus LAMS are needed for simple and complex pancreatic fluid collections.

## 7. Long-Term Outcomes

AP has morbidity during the attack itself, and that may be related to other organs’ involvement or from AP’s effect on the pancreas organ directly. Long-term outcomes post-AP are variable and not fully known. The risks from AP post-recovery can include, but are not limited to, abdominal and gastrointestinal symptoms, malabsorption or exocrine pancreatic insufficiency, recurrence of pancreatitis, and other factors that limit the quality of life. A substantial subset of patients have mild to moderate exocrine pancreatic insufficiency post AP (29%), with a smaller subset (6.2%) being diagnosed with severe exocrine pancreatic insufficiency post AP [53]. A systematic review showed that a quarter of all patients post-AP develop exocrine insufficiency during follow-up >36 months post-index admission. Alcoholic etiology and severe and necrotizing pancreatitis were associated with higher risks [54]. Endocrine complications can be substantial, as shown in observations that 15–30% of patients develop impaired glucose tolerance or diabetes within 1–3 years after even a single episode of AP [55,56]. Recent studies have shown that AP has long-term deleterious effects on physical health-related quality of life [57]. With most data on long-term follow up of AP in adults, similar studies in children are needed. The field will benefit from long-term observational studies in AP.

## 8. Clinical Trials and Research

There is a need for research that advances the prevention and treatment of AP and further understand its long-term sequalae. In a recent National Institute of Health workshop that identified unmet needs and areas needing accelerated focus in pancreatitis, multiple elements pertaining to AP were identified. [24] In AP, there remains a need for defining targeted clinical outcomes, as those can be patient-specific, like patient-reported outcomes, provider-subjective, or observer-reported outcomes, as well as performance outcome measures. Furthermore, predicting the development of severe AP and development of complications is needed, as it will allow risk-stratified management rather than an all comers-based treatment. In adults, there are few active interventional trials in AP, and much less are currently being performed in children.

## 9. Conclusions

With the prominent burden of AP amongst gastrointestinal diseases, the diagnosis and treatment of AP remains a top priority among pediatric and adult gastroenterologists. Table 1 summarizes comparison of epidemiology, diagnosis, contributing Factors, management, long term outcomes, and areas for research in pediatric and adult Patients.

There are many shared commonalities between pediatric and adult pancreatitis; however, it is important to appreciate the differences, even if sometimes nuanced, in order to make accurate diagnoses and initiate appropriate therapies. Pediatric gastroenterologists have benefited from the knowledge garnered in adults but are increasingly characterizing pediatric AP and initiating pediatric-specific research studies. Thus, there is hope that over the next decade we will see new therapeutic interventions that will improve the outcomes of those that suffer from AP, regardless of age.

## Figures and Tables

**Table 1:** Comparison of Acute Pancreatitis (AP) Epidemiology, Diagnosis, Contributing Factors, Management, Long Term Outcomes, and Areas for Research in Pediatric and Adult Patients.

Elements of Acute Pancreatitis	Pediatric	Adult
Epidemiology (See Figure 1A)	Incidence is stable.	Incidence may be decreasing. More common than in children.
Diagnosis	AP is defined by meeting two out of the three criteria: (i) abdominal pain and symptoms suggestive of pancreatitis (ii) lipase and/or amylase at least three times the upper limit of the normal range for the laboratory values used (iii) imaging findings of AP	
Etiologies/Contributing Factors (See Figure 1B)	Diverse	Gallstones and alcohol predominate
Management	Approximately 35% of patients with acute pancreatitis presenting to the Emergency Department will be discharged without admission. Foundational pillars of acute pancreatitis management: intravenous fluids, early enteral nutrition, and pain control. North American Society for Pediatric Gastroenterology, Hepatology, and Nutrition (NASPGHAN) and American Gastroenterology Association (AGA) with separate, but similar, guidelines in 2018 [30,31].	
Long-Term Outcomes	Limited data	25% of all patients with AP develop exocrine pancreatic insufficiency.
Research	Few interventional trials. Need for long-term observational cohort studies post AP. Increased need for etiology- and age-specific interventional trials for severity prediction and management of AP. Interventional trials underway for management of AP.	

## Data Availability

No new data were generated in this manuscript. Data sharing is not applicable to this article.
